# Ten recommendations to improve pharmacy practice in low and middle-income countries (LMICs)

**DOI:** 10.1186/s40545-020-00288-2

**Published:** 2021-01-06

**Authors:** Zaheer-Ud-Din Babar

**Affiliations:** Department of Pharmacy, Centre for Pharmaceutical Policy and Practice Research, University of Huddersfield, Queensgate, HD1 3DH Huddersfield UK

**Keywords:** Pharmacy, Pharmacy practice, Low and middle income countries (LMICs), Pharmaceutical systems

## Abstract

Medicines are important health interventions and their appropriate use could improve health outcomes. Throughout the globe, pharmacists play a very important role to improve the use of medicines. Though high-income countries are debating on futuristic approaches, independent prescribing of pharmacists, clinical skills, and to expand pharmacy services; a large majority of low and middle-income countries still lag behind to strengthen pharmacy practice. This paper presents a key set of recommendations that can improve pharmacy practice in low and middle-income countries (LMICs). The ten recommendations include (1) Mandatory presence of graduate-level pharmacists at community pharmacies (2) Clear demarcation of the roles and responsibilities of different categories of pharmacists (3) Effective categorization and implementation of medicines into (a) prescription medicines (b) pharmacists only medicines (c) over the counter medicines (4) Enforcement of laws and regulations for the sale of medicines (5) Prohibiting doctors from dispensing medicines (the dispensing separation between pharmacists and doctors). (6) Involving pharmacies and pharmacists in Universal Health Coverage Schemes to improve the affordability of medicines (7) Strengthening national medicines regulatory authorities to improve the quality, safety, and effectiveness of medicines (8) Training of pharmacists in clinical skills, vaccination, and minor ailment schemes (9) Promoting independent medicines information for consumers and healthcare professionals by developing national medicines information strategy (10) Mandatory Continuing Professional Development (CPD) programs for the Pharmacists.

## Introduction

Medicines are important health interventions and their appropriate use could improve health outcomes. Throughout the globe, pharmacists play a key role to improve the use of medicines. Though high-income countries debate on futuristic approaches; independent prescribing of pharmacists, clinical skills, and to expand pharmacy services [1] a large majority of low and middle-income countries still lag behind to strengthen pharmacy practice and to improve the “use of medicines” [2, 3].

It is important to note that low and middle-income countries (LMICs) are not a homogenous group, and there are large variations between them. Also, considerable progress in pharmacy has been made by some middle-income countries.

However, despite these variations, there are inherent commonalities and the following set of key recommendations could improve pharmacy practice in these countries. These recommendations are also increasingly vital in the context of the current Covid19 situation.

## 1. Mandatory presence of graduate-level pharmacists at community pharmacies

According to the laws and regulations, the mandatory presence of pharmacists at pharmacies is vital; however, in many LMICs, pharmacies are not always manned by the pharmacists [4, 5]. There could be several factors leading to the non-availability of graduate pharmacists including the perceived social status of pharmacists, organizational culture [6], the presence of equivalent diploma pharmacists (with the equivalent levels of authority), as well as the availability of fewer graduate pharmacists in countries. It has been noted from the literature that the presence of a graduate pharmacist is the single most decisive factor and this can improve the use of medicines in a country [4, 7].

In LMICs, a large number of pharmacies are non-pharmacist-run pharmacies (NPRPs). Having diploma pharmacists, who are not University graduates but have very similar powers and privileges to open pharmacies and medical stores. This confuses the patients and other healthcare professionals and erodes professionalism. The lay public is not able to distinguish between the different “categories” of pharmacists hence they consider them all the same.

If only graduate pharmacists are allowed to man pharmacies, this would help strengthen the pharmacy profession in LMICs. The compulsory presence of pharmacists at pharmacies in LMICs should be a priority, as it also paves the way for "extended pharmacy services".

## 2. Clear demarcation of the roles and responsibilities of different categories of pharmacists (a Graduate Pharmacist and a Diploma Pharmacist)

There is a need to have a clear demarcation of the roles and responsibilities of different classes of pharmacists. The graduate-level pharmacists should be in charge of running a pharmacy and dispensing medicines. If there are other categories of pharmacists in the country (diploma level pharmacists), this should be then clearly defined. There is a need to have a phase-wise abolishment of these pharmacists. Please note that the status of these pharmacists is very different from a pharmacy technician or a pharmacy assistant. As a pharmacy technician or an assistant works under the supervision of pharmacists, the diploma pharmacists can open medical stores and can dispense some classes of medicines.

However, due to the lack of effective enforcement of regulations, in reality, these diploma pharmacists dispense all classes of medicines. In LMICs, where the pharmacy profession is not well established, having a non-distinction between different classes of pharmacists further complicates the issue.

One argument given by authorities is not having the required numbers of graduate pharmacists in the country. This needs to be done with the increasing the number of graduate pharmacists as well as a phased reduction of diploma pharmacists. However, there are powerful lobbies with financial interests making it challenging to overcome this issue.

## 3. Effective categorization and implementation of medicines into (a) prescription medicines (b) pharmacists only medicines (c) over the counter medicines

It is important to categorize medicines into three different categories. This is the norm in highly developed western countries and LMICs can follow suit. Prescription medicines to be sold by prescription from doctors, pharmacists only (which the pharmacist can dispense these medicines without a prescription for minor ailments and third is over the counter (OTC)medicines.

Though in many LMICs there are laws and regulations in place for selling these medicines; however, there is a lax implementation of these laws. The sale of medicines or availability/non-availability (controlled availability of medicine) is a cornerstone of pharmacy practice in highly developed countries. This is where the authority of providing medicine to a patient makes pharmacist a credible healthcare professional. However, in many LMICs, a large number of medicines are available without a prescription [8]. This is not limited to certain classes of medicines but including a wide range of medicines including antibiotics [9].

Many pharmacists in LMICs also argue about having independent and supplementary prescribing roles for pharmacists. However, one needs to understand that the whole notion of independent and supplementary prescribing depends on giving pharmacists the authority to legally prescribe “some classes of medicines”. However, if in low-income countries, a large number of medicines are available without a prescription, then this exercise is indeed futile.

## 4. Enforcement of laws and regulations for the sale of medicines

If the laws and regulations are properly enforced in regards to the sale of medicines, this can greatly improve pharmacy practice. This could be done with political commitment as well as with effective regulation. In most countries, these laws are enforced through a drug regulatory authority or a drug control organisation.

The factors which could impact law enforcement include transparency, corruption, and myriad interests of healthcare professionals, pharmacy traders, and businesses. Also, in many LMICs, dispensing doctors can sell medicines, requiring effective regulation and control in this area.

## 5. Prohibiting doctors from dispensing medicines (the dispensing separation between pharmacists and doctors)

The dispensing separation between doctors and pharmacists is vital to improve the use of medicines [10]. Ideally, the doctors should prescribe medicines, while the pharmacist's role is to dispense. In a large majority of low and middle-income countries, doctors are still dispensing medicines. This is because of the economic benefits and the profits they earned through the sale of medicines. This is a conflict of interest as if the doctors have to make a profit by the sale of the medicines, then it may compromise their ability to prescribe medicines. Due to financial interests, they may dispense more medicines. Also, if a large majority of medicines are dispensed by doctors, pharmacists get very few medicines to dispense, limiting their ability to sustain businesses and pharmacies.

A systematic review of the literature comparing the practices of dispensing and non-dispensing doctors has shown that dispensing doctors prescribe more pharmaceutical items [11].

South Korea, Japan, and Taiwan have implemented the policy of separating the prescribing and dispensing roles for physicians and pharmacists [12]. One study showed that the number of drugs prescribed went down by about 4.7% after the policy was implemented in South Korea [13]. LMICs can learn lessons from these countries to improve practices in this area.

## 6. Involving pharmacies and pharmacists in Universal Health Coverage Schemes to improve the affordability of medicines

Many low and middle-income countries (LMICs) don’t have Universal Health Coverage Schemes similar to Australia, New Zealand, and the UK. It means that the pharmacies and general practices are not digitally connected and there are no co-payments from the government to subsidize medicines.

In many LMICs, the public sector provides free medicines to the patients; however, they have to pay for medicines at dispensing doctors' clinics and at private pharmacists. The research estimates that up to 70–90% of expenditures in LMICs are out of pocket [14].

The pharmacist’s role in improving the affordability of medicines is understudied. In many LMICs the government has started schemes to provide free services or some form of health insurance; however, there is a need to involve primary care doctors and pharmacies in these schemes. Hermansyah et al. [15] has explored the role of community pharmacy providing universal healthcare with some promising results in Indonesia; however, further work is needed on the issue.

The pharmacist’s role to promote affordability is also vital when consumers use generic medicines. This is done by providing advice and information to patients and consumers [16–18]. Generic medicines can save a large amount of money for patients, healthcare systems, and for society necessitating to build evidence-informed policies in this area [19–21].

## 7. Strengthening national medicines regulatory authorities to improve the quality, safety and effectiveness of medicines

Drug regulatory authorities' role is key to improve medicines quality, safety, and effectiveness of medicines in a country [22]. WHO has done a huge amount of work in this context, improving and strengthening medicines regulatory systems in LMICs [23].

Malaysia is one such example, which has played an excellent role to improve the quality, safety, and effectiveness of medicines [24]. This is being done by strengthening the drug regulatory authority in the country. The other LMICs need to learn from Malaysia's example. There is also some debate regarding improving consumers’ awareness with regards to medicines safety and counterfeit medicines. Though promoting awareness in society regarding counterfeit medicines is vital; however, this rarely works when a weak regulatory system is in place in a country.

## 8. Training of pharmacists in clinical skills, vaccination, and minor ailment schemes

There are challenges with regards to pharmacists' education and training across LMICs in clinical skills and in providing patient-oriented pharmacy services [25]. Although there are global professional standards for the provision of clinical pharmacy services; however, they are not always enforced in LMICs [26]. It is thought that pharmacists can play an increasingly important role in providing care in minor ailment schemes, chronic disease management, as well as in providing vaccination. However, a recent systematic review observed that though pharmacists can play an important role to increase access to vaccines yet evidence of their role in vaccinations remains limited across LMICs [27]. This demands further work on the topic as it can result in huge benefits for health systems and countries.

## 9. Promoting independent medicines information for consumers and healthcare professionals by developing national medicines information strategy

Providing independent medicines information to consumers and healthcare professionals is a global challenge [28]. This is also challenging with the increased information flow in the current internet age [29]. In high-income countries, Australia [30] and Finland [31] are two good examples. Finland [31] has been instrumental to develop a national medicines strategy, while Australia has successfully provided medicines information to consumers and healthcare professionals alike [30]. However, this is not uniform across the board in high-income countries and the challenges lay ahead. A survey conducted in eight European countries has shown that more efforts are warranted to develop evidence-based drug information [32].

In LMICs, the scale of the problem is large and there are enormous challenges in providing medicines information to consumers and healthcare professionals. This is coupled with lower levels of health literacy in consumers, as well as the training of pharmacists and physicians in the area of "independent objective medicines promotion". LMICs have enormous challenges related to irrational prescribing, fake news as well as wrong and misleading medicines information. Nevertheless, this is vital to promote independent information sources as they can improve patient health outcomes. The importance of these resources is also increasingly vital in the context of the current covid19 situation.

## 10. Mandatory Continuing Professional Development (CPD) programs for the Pharmacists

Low and Middle-Income Countries (LMICs) face challenges both in terms of numbers of pharmacists as well as education and training. According to the International Pharmaceutical Federation (FIP), though pharmacists numbers have increased globally; however, much of this growth is in the World Health Organization’s Eastern Mediterranean Region and in the European regions. Growth in pharmacist capacity was lowest in low-income countries and the African region [33]. However, differences between high-income countries and LMICs are not only quantitative (in terms of numbers of pharmacists). There are huge gaps and the differences, the way pharmacy is practiced across the globe [34].

Though the need for advanced pharmacy education is recognized globally; however, in many LMICs, there is limited capacity and experience to develop continuing professional development (CPD) [35–37]. In this context, LMICs must develop a mandatory CPD model for pharmacists that can update and advance and update their training and skills. Also, the pharmacy system strengthening and its role in improving clinical pharmacy practice should be a necessary component of CPD in LMICs [38].
